# The Sleepy Teenager – Diagnostic Challenges

**DOI:** 10.3389/fneur.2014.00140

**Published:** 2014-08-04

**Authors:** Anne-Marie Landtblom, Maria Engström

**Affiliations:** ^1^Department of Clinical and Experimental Medicine, Division of Neurology, IKE, County Council, Linköping University, Linköping, Sweden; ^2^Neurology Unit, Department of Medical Specialist, IMM, County Council, Linköping University, Motala, Sweden; ^3^Department of Neuroscience, Uppsala University, Uppsala, Sweden; ^4^Division of Radiological Sciences, Department of Medical and Health Sciences, Linköping University, Linköping, Sweden

**Keywords:** adolescent, narcolepsy, hypersomnia, delayed sleep phase syndrome, computer gaming, fatigue, cognition disorders, Kleine–Levin syndrome

## Abstract

The sleepy teenager puts the doctor in a, often tricky, situation where it must be decided if we deal with normal physiology or if we should suspect pathological conditions. What medical investigations are proper to consider? What differential diagnoses should be considered in the first place? And what tools do we actually have? The symptoms and problems that usually are presented at the clinical visit can be both of medical and psychosocial character – and actually they are often a mixture of both. Subsequently, the challenge to investigate the sleepy teenager often includes the examination of a complex behavioral pattern. It is important to train and develop diagnostic skills and to realize that the physiological or pathological conditions that can cause the symptoms may have different explanations. Research in sleep disorders has shown different pathological mechanisms congruent with the variations in the clinical picture. There are probably also different patterns of involved neuronal circuits although common pathways may exist. The whole picture remains to be drawn in this interesting and challenging area.

## Physiology Versus Pathology in Adolescent Sleep

Healthy youngsters often demonstrate a change of sleeping behavior, including longer sleep periods and a tendency to delayed sleep phase. This can be explained by physiology. Among the many behavioral changes associated with adolescent development are subsequently later bedtimes, which, however, also can result in less sleeping hours if the person has to get up early. In healthy adolescent sleep, one can identify a maturation of both the homeostatic and circadian processes regulating sleep. Some authors describe how these maturational changes in combination push adolescents toward later bedtimes, while societal demands, such as early school start times, result in a pattern of insufficient and ill-timed sleep (1). The implications of sleep curtailment during this developmental period are of great importance.

It seems crucial to expand the knowledge on how these physiological circumstances sometimes may facilitate a condition bordering pathology. The question is if and how such a sleeping behavior may transform into a delayed sleep phase syndrome (DSPS). This is a condition that is not always accompanied with direct harmful somatic effects but must be taken seriously and regarded as a pathological condition, because of its potential to cause substantial impact on the social life as well as psychological or even psychiatric problems. DSPS has been shown to be a forerunner to psychiatric distress (2).

### Social perspective

In this context of physiology versus pathology, it is also interesting to regard DSPS from the social perspective. The central question is if a more flexible attitude toward working schedules may reduce or inhibit the negative effects of DSPS. Therapy has become increasingly integral to the rhythm of everyday life, particularly in the western world, where medical treatment and pharmaceutical consumption have become a “means for normalizing oneself to social expectations,” according to one author of an interesting article on this theme (3). He explains his view like: “I draw on fieldwork with people who experience sleep disorders – narcolepsy, sleep apnea, and delayed and advance sleep phase syndrome – to explicate models of treatment and consider how these medical spatiotemporalities formulate emergent everyday orders of life.” One might consider a potential connection of sleep phase disturbances and unemployment among young in the western world. Another recent article with a bearing on this issue is a Norwegian study that analyses the role of school start in the morning hours (4). A later school start times may both lengthen nocturnal sleep and increase students’ alertness in early morning.

In addition to such potential effects of adolescent sleep physiology, the modern context offers an environment with computer games, social media, and availability of entertainment around the clock in combination with electric light that may disturb the human internal clock in terms of the circadian rhythms.

### Computer gaming and sleep

A lot of young people, and more male than female, exhibit excessive nightly use of computer games and some of these persons perceive problems and interference with their social life (5). Many demonstrate sleep disturbances but the question is how frequent this develops into a regular DSPS. The sex ratio in DSPS is still reported to be 50/50% for men and women even with a slight predominance of females (6) with the exception of one study from Asia (2) where cultural factors may have played a role. Also, milder forms of sleep phase disturbances can motivate investigation focusing the potential risk with frequent use of social media and entertainment in the night. It is also important to highlight the risk that such young men can succumb to diseases and with the obvious risk of not being investigated or diagnosed.

Youth with disturbances of sleep after computer gaming often have a long sleep period during the day, but with fragmentation and poor sleep efficiency. Research findings diverge regarding the degree of addiction that can emerge through computer gaming – it seems to be individual and has been related to certain personality types. Young persons who play computer games at night have been shown to have statistically significant more frequent depression (7, 8). This tendency to depression is not correlated to the amount of hours that are spent with the game but to the circadian rhythm, i.e., if the persons play at night. It has been observed that the critical hour for younger children is earlier than for older children/teenagers. It is important to investigate if there is a concomitant depression. One study has even shown a connection between computer gaming and suicide (9). Some studies support that the risks from computer games increase when there is a frequent concomitant usage of cell phones. Differences between the sexes regarding the impact of these risks have been shown.

### Sleepy teenager – differential diagnostics

Probably, there is a substantial amount of young persons with increased sleepiness who do not search health care, because it is generally accepted in the society that a teenager is sleepy. This notion may also contain prejudice that sometimes is directed toward the person, indicating that the sleepiness is a negative side of the personal character. Sloppiness, not trying enough, not taking school studies or work seriously, having a bad character are common views that accompany tiredness in a young person. It is true that teenagers can have several reasons for being tired from a physiological perspective: the processes of puberty, increasing body length, sometimes rapidly growing, personal conflicts that arise from new demands in the family or at school, psychological problems in their role seeking, and so forth, are examples of causes that may explain an increasing tiredness.

Diseases that must be brought to mind for all persons that work professionally with teenagers, are obstructive airway syndrome with *obstructive sleep apnea syndrome* (OSAS), *narcolepsy, hypersomnia*, especially the *Kleine–Levin syndrome* (KLS), *fatigue* due to somatic disease, especially early multiple sclerosis (MS), which can start without obvious focal neurological dysfunction. Other diseases that can cause fatigue and start in young ages are for example rheumatoid arthritis, allergy, Mb Crohn, and ulcerative colitis. The increase in narcolepsy incidence during 2010 and 2011 after the Pandemrix vaccination against the H1N1 virus seen in children and young people in many countries motivates specific focus on this disease (10, 11). New cases with mild pathology are still diagnosed in Sweden, supporting the importance of keeping this disease in mind. Of course, a potential *depression* or the use of *narcotic drugs* must also be excluded.

It is important to consider investigation of sleep when there are problems with cognition demonstrated by performance issues at school. Some of the conditions mentioned above may have an impact on cognition, for example clearly demonstrated as working memory problems in KLS (12–17) and cognitive dysfunction in MS, often due to fatigue (18, 19). An effect on sleep and cognition has also been demonstrated in children with frequent computer gaming (20). Of course, lack of sleep can have an impact on cognition as well, and it is important to consider the possibility of sleep deprivation or sleep restriction.

## Differential Diagnostics and Pathological Background

Among all young people with sleep disturbances it is of great importance to reflect over the clinical differences between physiological and pathological conditions, in order to be able to recognize the sleep disorders. We must decide the diagnosis, give timely information and treat the symptoms.

Obstructive sleep apnea, narcolepsy, and periodic hypersomnia like in the KLS as well as fatigue due to chronic diseases are such conditions important to learn to detect, especially for general practitioners, pediatricians, and school health workers.

### Narcolepsy

*Background: Narcolepsy is caused by neuron loss of orexin-producing neurons in the lateral hypothalamus*.

In narcolepsy, it is important to investigate the day time sleepiness, which usually is excessive. This can be done with the instrument Epworth sleepiness scale (ESS). Cataplexy, i.e., loss of tonus mostly in emotional situations, especially in laughter and joking, but also in sports or sex, must be investigated. It is important to remember that the association to emotions is not strictly necessary – there are patients who demonstrate a varying sloppiness in the face or extremities, mostly seen children. The cataplectic face, with protruding tongue and facial weakness, has been focused recently because reduced connections to emotional stimuli has caused problems to identify correct diagnosis. Other typical symptoms are hypnagogic/hypnopompic hallucinations, sleep paralysis, weight gain, disturbed night sleep, and nightmares. In children, there may also appear pubertas praecox. Laboratory tests like HLA and spinal orexin as well as neurophysiological tests are frequently part of the investigation.

### Kleine–Levin syndrome

*Background: Kleine–Levin syndrome has been connected to asymmetric thalamic dysfunction and hypoperfusion of temporal and frontal areas*.

**Figure 1:**
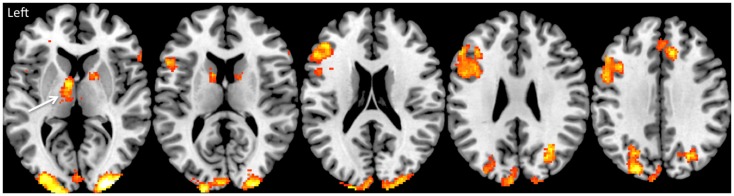
**Brain activation during working memory performance in the Kleine–Levin syndrome**. This shows typical brain activation as measured by fMRI in the fronto-parietal executive network in a group of 18 KLS patients. The arrow shows pathological hyperactivation (BOLD activity) of the left thalamus.

The KLS is a rare form of hypersomnia (21) that must be investigated through a survey of the periodicity, which is crucial. The periodicity can involve sleep, but also psychological discomfort and cognitive dysfunction, especially after some years of disease. Periods are sometimes very regular, for example 1 week/month. Associated symptoms are hyperphagia (or even loss of appetite), depersonalization, derealization, regression, aggression, communication problems, and subjective “vertigo,” the feeling of “Being in a bubble.” Hypersexuality can be a part of the behavioral disturbance observed but is not obligatory for the diagnosis. Imaging with single-photon emission computed tomography (SPECT) has revealed asymmetric thalamic dysfunction in episodes (22) and interepisode hypoperfusion of temporal and frontal areas (12, 13). Between episodes functional magnetic resonance imaging (fMRI) has revealed thalamic dysfunction on a group level (23, 24), see Figure 1.

Because of the difficulty to diagnose this disorder, there are probably many undiagnosed cases. Investigation with neuropsychological testing in search for interperiodic disturbances of the working memory as well as interperiodic cerebral blood flow analysis (CBF) using SPECT have been suggested by our research group (12, 13).

In general, to investigate daytime sleepiness or hypersomnia one should use the possibility to survey the sleep pattern with neurophysiological methods like polysomnography, actigraphy, and multiple sleep latency test. Here, it is possible to identify conditions like OSAS, to find the REM sleep aberrations that are typical for narcolepsy and to measure sleep length and depth in order to diagnose hypersomnia. Idiopathic hypersomnia is believed to be a quite rare condition, so efforts must be made in order to exclude other conditions.

### Fatigue in MS

*Background: Injured neuronal circuits due to white matter lesions cause connectivity disruption and functional reorganization, which in turn leads to reduced efficiency of networks. The pathological immune response may contribute to fatigue*.

Fatigue in MS is a very frequent symptom and may be mixed up with normal sleepiness in young people or with exhaustion due to depression (25). It is consequently necessary to screen for focal neurological symptoms and perform a thorough neurological examination in all sleepy teenagers. It is also important to remember that focal findings are *not* obligatory for a MS diagnosis. Common symptoms are sensory disturbances, micturition problems, but also heat sensitivity (26) and cognitive problems. An MRI of the brain may be necessary to investigate typical white matter lesions. Research involving fMRI have resulted in hypotheses describing potentially injured neuronal circuits due to white matter lesions causing connectivity disruption and functional reorganization, which in turn lead to reduced efficiency of networks – to longer neuronal paths and increased energy consumption (27, 28). There are now indications that the thalamo-striato-cortical network can be involved (19). There may also be damage of tracts associated to alertness like the reticular formation. In addition, a pathological immune response has been proposed as a plausible factor (29).

### Delayed sleep phase syndrome

*Background: Delayed sleep phase syndrome is a chronobiological disorder caused by a delay in the circadian clock in the nucleus suprachiasmaticus. A delayed melatonin rhythm supports the involvement of the pineal gland. There seems to be a genetic component (polymorphisms in the clock genes) for some patients*.

Delayed sleep phase syndrome can be primarily organic, with a disturbance in the melatonin secretion, or secondary, due to psychological factors. It can also have a connection to an attention-deficit/hyperactivity disorder (ADHD) (30). Investigation can be made by actigraphy, showing the disturbance of sleep phase and sometimes the melatonin peak is measured (31). Treatment that can be tried is melatonin and light exposure. Cognitive behavioral therapy has also been suggested. Research on clock genes has revealed that a polymorphism in the clock gene PER3 may lie behind melatonin inhibition and an alerting response to blue-enriched light (32). Old and new studies demonstrate a possible link between DSPS and personality disorders, which support the hypothesis that inborn details in the sleep–wake rhythm lead to specific symptoms characteristic for personality disorders in the social and functional domain (33). Recent research indicates that people with DSPS more often develop anxiety, depression, and substance use. Interestingly, this was also true for persons with an evening-type circadian preference (34).

### Depressive disorders

*Background: Vegetative symptoms, especially hyposomnia, can occur in depression. REM sleep is often functionally deficient, which can increase psychological symptoms like helplessness and mood disturbance. However, also hypersomnia is in fact a possible depressive symptom, for example in atypical depression. Increased sleep can in addition be a side effect of antidepressive drugs and also a daytime consequence of early awakenings*.

Depression is often accompanied by hyposomnia and disturbance of REM sleep has been reported. Typically, depressive disorders are associated with early awakenings in combination with increased psychological discomfort. Voluntary sleep deprivation has been found to be beneficial in depression (35). Other common vegetative symptoms in depression are besides hyposomnia decreased appetite and weight loss. But there are also depressive conditions with so called reversed vegetative symptoms, i.e., hyperphagia, weight gain, increased sexual drive, and hypersomnia that have been defined as “atypical depression.” During the last decade, however, this perception has been questioned (36). Day time sleepiness in typical depression may also be secondary to early awakenings. Finally, some antidepressive medications can give sleepiness as a rather frequent side effect that even sometimes is beneficial when hyposomnia is a prominent symptom.

### Use of narcotic drugs

*Background: Use of narcotic substances can affect sleep in various ways. Sedating drugs like cannabis can give somnolence, and activating drugs like amphetamine can cause sleepiness after extended awake periods*.

Laboratory tests for analysis of narcotic substances should be a part of the investigation of the sleepy teenager, because the patient may not reveal substance use for legal or other reasons. Cannabis is one of the most important substances in this respect. One study of chronic users showed that higher THC and 11-OH-THC concentrations were significantly associated to shorter sleep latency, less difficulty falling asleep, and more daytime sleep the following day (37). However, nighttime sleep tended to decrease, which can be a sign of tolerance to the somnolent effects in chronic users. Besides the fact that substances can affect mood and alertness in different ways, sedating or activating, there are interesting new findings regarding brain physiology and drug addiction. The possible relationship between habenula, a small structure located posterior to the thalamus and adjacent to the third ventricle, and drug abuse has recently been focused (38). The habenula receives inputs from the limbic system and basal ganglia. Through a process involving GABAergic cells that leads to GABA inactivating dopaminergic cells, the habenula controls dopamine levels in the striatum and plays a critical role in the rewarding systems. It also modulates serotonin levels, norepinephrine and acetylcholine release and subsequently influences the brain’s response to pain, stress, sleep, and reward. Besides a probable relationship to drug abuse, dysfunction of the habenula has also been linked to depression and schizophrenia.

## Functional Diagnostics

Today, the diagnostic tools in sleep disorders consist of clinical assessment, laboratorial, and neurophysiological investigations. Radiology only demonstrates suggestive results that might be developed in the future. Patients with hypersomnia or fatigue/day time sleepiness seldom have morphological findings on conventional radiological investigations, if they do not suffer from a secondary condition. But there are obvious functional disturbances connected to the symptoms. Functional imaging (SPECT and fMRI) in narcolepsy and KLS constitute examples that contain future diagnostic possibilities as stated above. It is, however, important to point out that pathological findings reported in some sleep disorders not yet have been thoroughly investigated and compared in other disorders. For example, the disturbance that our group identified regarding working memory in interperiodic KLS was detected as a difference from the standard. This may theoretically, as well be present in other disorders like narcolepsy or DSPS. Also, the presence of hypoperfusion in SPECT in temporal and frontal parts of the brain that we saw in KLS in about half of all cases (Vigren et al., unpublished), may not be a specific finding for this disorder, but may well exist also in other conditions with sleep aberrations. Consequently, a lot of interesting investigations remain to be done and there is enough basic knowledge to design valuable comparative research. For fMRI, the challenge is to transform methods for investigations of patient groups into paradigms that allow us to measure individuals with a diagnostic purpose.

Functional imaging diagnostics is a promising method in these respects. There is, however, a need for more research before it is possible to introduce these methods into clinical praxis.

In conclusion, the sleepy teenager should be investigated with a personal interest using professional skills regarding many diseases that usually are connected to a variety of different medical subdisciplines. The common symptom of “sleepiness” must in fact be divided into more specific terms like excessive daytime sleepiness, hypersomnia, periodic hypersomnia, fatigue (mental or somatic), or delayed sleep phase. One should also consider the potential presence of nightly hyposomnia, insomnia, and early awakenings, factors that can have an impact on the alertness during the day. Obviously, a thorough medical history must be taken, and because some diseases are accompanied by amnesia or working memory problems, this history should be taken together with parents or co-habitant persons. In addition to a detailed medical history and a somatic/neurological examination, the initial investigation can gain invaluable information from a sleep diary, specific scoring instruments like ESS and HAD (Hospital Anxiety and Depression score) and an actigraphy.

## Conflict of Interest Statement

The authors declare that the research was conducted in the absence of any commercial or financial relationships that could be construed as a potential conflict of interest.
